# Fracture patterns and causes in the craniofacial region: an 8-year review of 2076 patients

**DOI:** 10.1186/s40902-018-0168-y

**Published:** 2018-10-15

**Authors:** Ki-Su Jin, Ho Lee, Jun-Bae Sohn, Yoon-Sic Han, Da-Un Jung, Hye-Young Sim, Hee-Sun Kim

**Affiliations:** 1 Department of Oral and Maxillofacial Surgery, Seoul Metropolitan Government-Seoul National University Boramae Medical Center, 20 Boramae-ro 5 Gil, Dongjak-gu, Seoul, 07061 Republic of Korea; 2 Section of Dentistry, Seoul Metropolitan Government-Seoul National University Boramae Medical Center, Seoul, Republic of Korea

**Keywords:** Fracture, Maxillofacial trauma, Etiology, Incidence

## Abstract

**Background:**

For proper recovery from craniofacial fracture, it is necessary to establish guidelines based on trends. This study aimed to analyze the patterns and causes of craniofacial fractures.

**Methods:**

This retrospective study analyzed patients who underwent surgery for craniofacial fractures between 2010 and 2017 at a single center. Several parameters, including time of injury, region and cause of fracture, alcohol intoxication, time from injury to surgery, hospitalization period, and postoperative complications, were evaluated.

**Results:**

This study analyzed 2708 fracture lesions of 2076 patients, among whom males aged 10 to 39 years were the most numerous. The number of patients was significantly higher in the middle of a month. The most common fractures were a nasal bone fracture. The most common causes of fracture were ground accidents and personal assault, which tended to frequently cause more nasal bone fracture than other fractures. Traffic accidents and high falls tended to cause zygomatic arch and maxillary wall fractures more frequently. Postoperative complications—observed in 126 patients—had a significant relationship with the end of a month, mandible or panfacial fracture, and traffic accidents.

**Conclusions:**

The present findings on long-term craniofacial fracture trends should be considered by clinicians dealing with fractures and could be useful for policy decisions.

**Electronic supplementary material:**

The online version of this article (10.1186/s40902-018-0168-y) contains supplementary material, which is available to authorized users.

## Background

The craniofacial area plays an important role not only in function but also in esthetics. Therefore, craniofacial fractures due to trauma cause functional and esthetic problems and may threaten the airway maintenance in severe cases. They can also result in permanent functional and esthetic damage, depending on the outcome of treatment. Such damage also affects social activities and may result in economic and psychological problems [1, 2].

To enable efficient first aid and achieve desirable recovery from craniofacial injury, it is necessary to analyze the patterns and causes of craniofacial fracture and establish appropriate guidelines based on the findings. In addition, statistical data related to trauma patterns are essential indicators in determining policies related to trauma, placement of emergency workforces, and for the calculation of related insurance costs. There have been many studies in this regard so far; however, owing to the diversity of survey methods and ethnic, social, geographic, and age differences among the study populations, these studies have shown varying results [3–6]. Therefore, there is a need for continuous updating of fracture patterns and causes.

This study conducted clinical and statistical analyses on craniofacial fractures in the past 8 years. The purpose of this study was to update the information on fracture patterns and causes in the capital region of a Northeast Asian country. The specific aims of this study were to reveal the relationship between the time of injury and number of patients, to analyze the correlation of fracture cause and region, and to determine the risk factors for postoperative complications.

## Methods

This single-center, retrospective, observational study investigated the medical records of all patients who had undergone surgery for cranial and facial bone fractures between January 2010 and December 2017 at a secondary care hospital. For fractures of the cranial and midfacial bones and mandible, only those patients who underwent closed reduction or open reduction and internal fixation in the operation room were included in the analysis. Patients treated with conservative management in the outpatient department were excluded. The investigated parameters were sex, age, diabetes mellitus, smoking status, time of injury (day of the week/month/year), region and cause of fracture, alcohol intoxication at the time of the injury, time from injury to surgery, hospitalization period, and postoperative complications. The causes of fracture were classified into the following six categories: ground accident, slipping on the ground and hitting the floor or nearby objects; fall, fall from a height that one’s feet could not reach; flying object, hit by a flying object; assault, intentionally or unintentionally hit by another person; sports activity, accidents during sports activities; and traffic accident, accidents involving automobiles. Surgery was performed by a specialist with at least 5 years of clinical experience in oral and maxillofacial surgery, plastic surgery, otolaryngology, ophthalmology, or neurosurgery. Medical records were reviewed by two surgeons, each with 3 years of clinical experience as oral and maxillofacial surgeons. The need for informed consent was waived. This study was approved by the institutional review board and complied with the tenets of the 1964 Declaration of Helsinki and its later amendments.

### Variables

In the analysis of seasonal distribution, the primary predictor variable was the date of injury, and the primary outcome variable was the number of fracture patients. In the analysis of fracture cause, the primary predictor variable was the cause of fracture, and the primary outcome variable was the region of fracture. In the analysis of postoperative complications, the primary predictor variables were sex, age, diabetes mellitus, smoking, alcohol intoxication, day of the week, beginning/middle/end of the month, time from injury to treatment, duration of admission, fracture site, and fracture cause; the primary outcome variable was the occurrence of postoperative complication.

### Statistical analysis

Continuous variables are expressed as mean ± standard deviation, and categorical variables are expressed as number or number (percentage). The time of occurrence of fracture was analyzed by analysis of variance or the Kruskal–Wallis test. Friedmann’s test and the Wilcoxon signed-rank test were used to determine the distribution of left- and right side fractures. Correlation analysis between the fracture cause and region was determined using Pearson’s chi–square test followed by multiple comparisons using the Bonferroni method. Logistic regression analysis was performed to identify the factors that affected postoperative complications; variables with statistical significance in univariable analysis were further subjected to multivariable analysis. Differences were considered statistically significant at *p* values < 0.05, and all analyses were performed using the IBM SPSS software (version 22.0; IBM Corp., Armonk, NY, USA).

## Results

### Demographic and seasonal distribution of patients with fractures

**Fig. 1:**
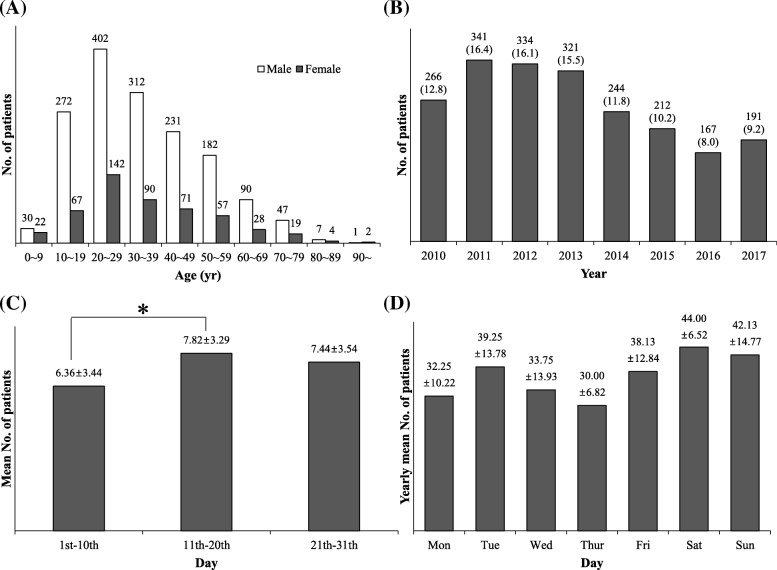
Demographic and seasonal distribution of patients with craniofacial fractures. **a** Sex and age. **b** Annual incidence. **c** Mean patient number of each trisection of a month. Each month was divided into the first, middle, and last trisections. **d** Weekly incidence. *Statistical significance, *p* < 0.05 (by analysis of variance). Values are presented as number (%) or number only

This study analyzed 2708 fracture lesions of 2076 patients, among whom men were more numerous than women (75.8%). In both sexes, patients in their twenties and thirties were more numerous than those in other age groups (Fig. 1a). Compared to 2010, the incidence of fracture had increased in 2011 but has decreased since then (Fig. 1b). The monthly distribution of the incidence of fracture was fairly even from January to December. Among the first, middle, and last trisections of each month, the number of patients was greater in the middle trisection than in the first (*p* = 0.025) (Fig. 1c). The numbers of patients with fracture tended to be greater on Saturdays and Sundays than on other days of the week, although there was no statistically significant difference in this regard (Fig. 1d).

### Region of fracture

**Fig. 2:**
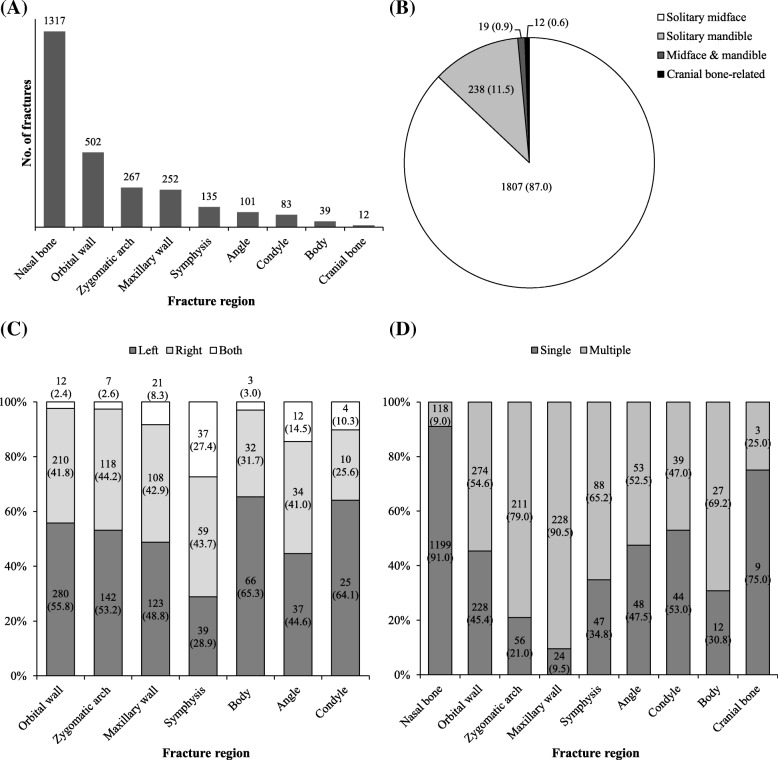
Region of fracture. **a** Distribution according to the craniofacial region. **b** Distribution among three parts of the . **c** Left and right distribution in each region. **d** Ratio of single-region and simultaneous fractures. Symphysis, mandibular symphysis or parasymphysis; Angle, mandibular angle; Condyle, mandibular condyle; Body, mandibular body. Values are presented as number (%)

The nasal bone was the most common region of fracture (48.6%), followed by the orbital wall and zygomatic arch (Fig. 2a). The most common fracture type was a solitary midfacial fracture (87.0%), followed by solitary mandible fracture (Fig. 2b). In the analysis of laterality of fractures (excluding those of the nasal bone and cranial bone), unilateral fractures were found to be more common than bilateral fractures (*p* = 0.002), with left side fractures being more common than right side fractures, although the difference was not statistically significant (Fig. 2c and Fig. 4a). Upon analysis of the overall ratio of single and multiple fractures, 80.2% of patients were found to have single fractures. However, in terms of individual regions, the rates of single fracture were higher than those of simultaneous fracture only in the nasal bone, cranial bone, and mandibular condyle (single fracture, 91.0%, 75.0%, and 53.0%, respectively). At other fracture sites—such as the maxillary wall and mandibular body—the rates of single fracture were lower than those of simultaneous fracture (single fracture, 9.5% and 30.8%, respectively) (Fig. 2d). In terms of individual causes, the rates of simultaneous fracture were relatively higher in fractures associated with fall and traffic accident than other causes (simultaneous fracture, 43.5% and 38.0%, respectively, Fig. 4b). Additional file 1: Table S1 shows the distribution of specific fracture regions among the study population.

### Cause of fracture

**Fig. 3:**
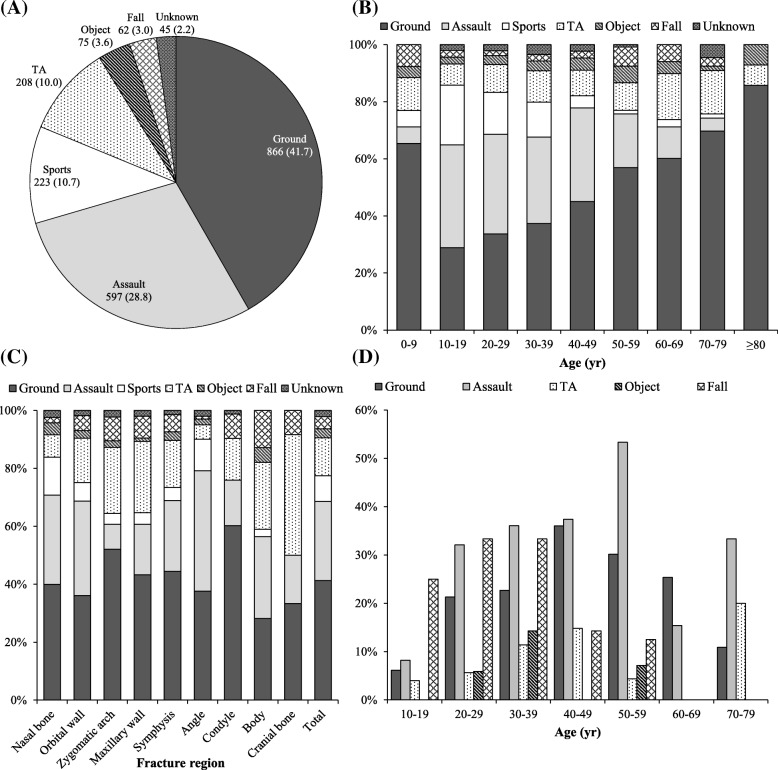
Cause of fracture. **a** Overall distribution. **b** Distribution according to age group. **c** Distribution according to fracture region. **d** Incidence of alcohol intoxication in each fracture cause, distributed according to age group. Ground, ground accident; TA, traffic accident; Object, hit by a flying object; Symphysis, mandibular symphysis or parasymphysis; Angle, mandibular angle; Condyle, mandibular condyle; Body, mandibular body. Values are presented as number (%)

**Fig. 4:**
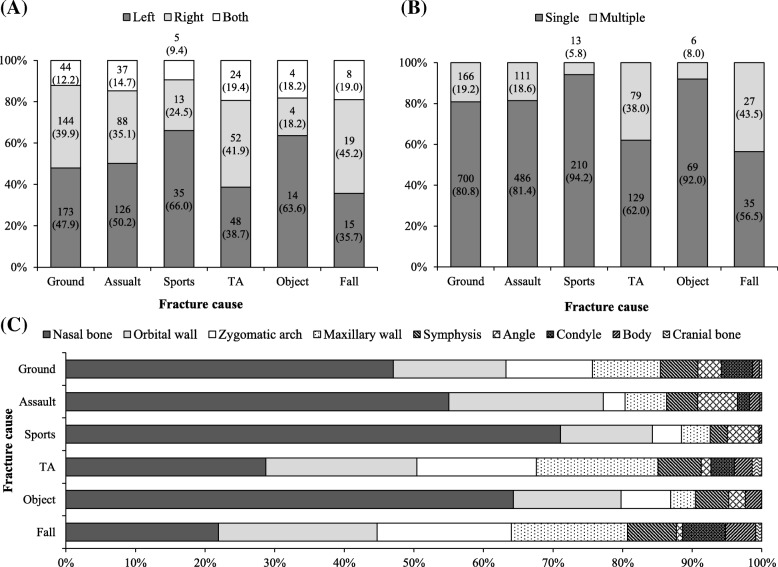
Fracture types according to the fracture cause. **a** Left and right distribution in each cause. Patients with solitary nasal bone or cranial bone fracture were excluded. **b** Ratio of single-region and simultaneous fractures. **c** Distribution of fracture region according to fracture cause. Ground, ground accident; TA, traffic accident; Object, hit by a flying object; Symphysis, mandibular symphysis or parasymphysis; Angle, mandibular angle; Condyle, mandibular condyle; Body, mandibular body. Values are presented as number (%)

Ground accident was the most common cause of fracture (41.7%), followed by assault and sports activity (Fig. 3a). However, in the age group analysis, the most common cause of fracture among teenagers and patients in their twenties was found to be assault (36.0% and 34.9%, respectively), followed by ground accident (28.9% and 33.6%, respectively) (Fig. 3b). Furthermore, according to the fracture region, the most common cause of zygomatic arch and maxillary wall fractures was ground accident (52.1% and 43.3%, respectively), followed by traffic accident (22.8% and 24.6%, respectively). The most common cause of mandibular angle fracture was assault (41.6%), followed by ground accident (37.6%) (Fig. 3c). Fracture associated with alcohol intoxication was observed in 415 patients (20.0%). Upon analysis of incidence of alcohol intoxication according to the fracture cause (excluding sports activity and unknown cause) and age group, fall was found to be the most common cause for alcohol-related fracture among teenagers and patients in their twenties (25.0% and 33.3%, respectively), followed by assault (8.2% and 32.1%, respectively). However, among patients in their thirties to seventies, except those in their sixties, assault was the most common cause for alcohol-related fracture (30–39, 40–49, 50–59, and 70–79 years, 36.1%, 37.4%, 53.3%, and 33.3%, respectively) (Fig. 3d). Upon correlation analysis of the cause and region of fracture, relative to fractures at other sites, the nasal bone fracture was found to have a greater causal relationship with fracture due to the ground accident, assault, sports activity, and flying object. In addition, zygomatic arch and maxillary wall fractures had a greater causal relationship with fracture due to traffic accident and fall than fractures at other sites. In other words, among the six fracture causes, ground accident, assault, sports activity, and flying object tended to cause nasal bone fractures more frequently than other types of fractures, with traffic accident and fall tending to cause zygomatic arch and maxillary wall fractures more frequently than other types of fractures (Fig. 4c and Table 1).

**Table 1:** Correlation of fracture cause with fracture region

Etiology	Region of fracture									Total
	Nasal bone	Orbital wall	Zygomatic arch	Maxillary wall	Symphysis	Angle	Condyle	Body	Cranial bone	
Ground	526 (47.0)*	181 (16.2)	139 (12.4)	109 (9.7)	60 (5.4)	38 (3.4)	50 (4.5)	11 (1.0)	4 (0.4)	1118
Assault	406 (55.0)*	164 (22.2)	23 (3.1)	44 (6.0)	33 (4.5)	42 (5.7)	13 (1.8)	11 (1.5)	2 (0.3)	738
Sports	172 (71.1)*	32 (13.2)	10 (4.1)	10 (4.1)	6 (2.5)	11 (4.5)	0 (0)	1 (0.4)	0 (0)	242
TA	102 (28.7)	77 (21.7)	61 (17.2)*	62 (17.5)*	22 (6.2)	5 (1.4)	12 (3.4)	9 (2.5)	5 (1.4)	355
Flying object	54 (64.3)*	13 (15.5)	6 (7.1)	3 (3.5)	4 (4.8)	2 (2.4)	0 (0)	2 (2.4)	0 (0)	84
Fall	25 (21.9)	26 (22.8)	22 (19.3)*	19 (16.7)*	8 (7.0)	1 (0.9)	7 (6.1)	5 (4.4)	1 (0.9)	114
Total	1285 (48.5)	493 (18.6)	261 (9.8)	247 (9.3)	133 (5.0)	99 (3.7)	82 (3.1)	39 (1.5)	12 (0.5)	2651

Fifty-seven cases of unknown etiology were excluded

Values are presented as number (%) or number only

The sum of the percentage value does not equal 100% because of rounding

*Ground ground accident, TA* traffic accident, *Symphysis* mandibular symphysis or parasymphysis, *Angle* mandibular angle, *Condyle* mandibular condyle, *Body* mandibular body

*Statistical significance, *p* < .05 (by Pearson’s chi–square test followed by multiple comparisons using the Bonferroni method)

### Time course

**Fig. 5:**
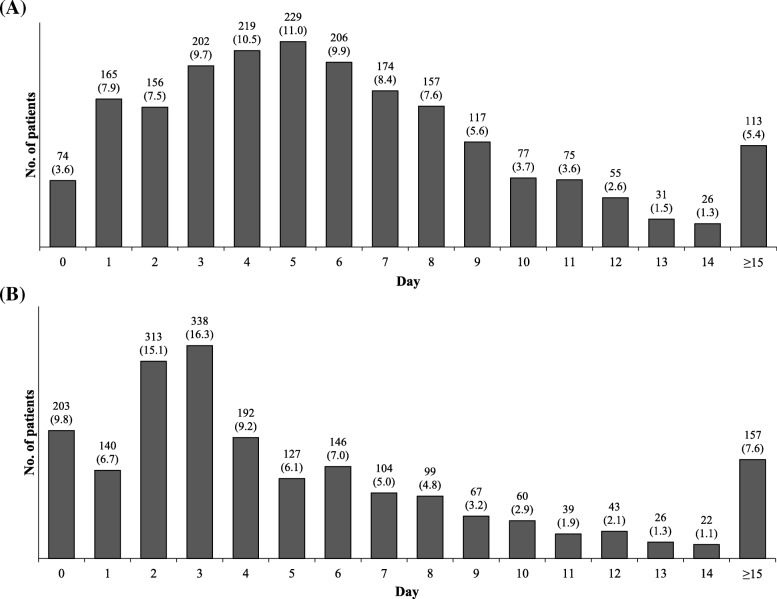
Time course. **a** Time from injury to surgery. **b** Hospitalization period. Values are presented as number (%)

In most cases, the interval between injury and surgery was 4 to 5 days, with 68.6% of the patients undergoing surgery within a week of injury (Fig. 5a). Most patients were hospitalized for a total period of 2 to 3 days. Most patients (75.3%) were discharged within a week of hospitalization (Fig. 5b).

### Postoperative complications

Postoperative complications were observed in 126 patients (6.1%). Nerve damage was the most common complication (72.0%), followed by infection and malocclusion (Tables 2 and 3). The results of multivariable logistic regression analysis showed a meaningful association between postoperative complications and the last trisection of the month, solitary mandibular fracture, simultaneous midfacial and mandibular fracture, and traffic accidents (Table 4). The results of the univariable logistic analysis are shown in Additional file 1: Table S2.

**Table 2:** Postoperative complications according to the fracture region

Complication	Nasal bone	Orbital wall	Zygomatic arch	Maxillary wall	Mandible	Total
Nerve damage	2		54*		39	95
Infection	2	1	–	5	10	18
Malocclusion	–	–	–	–	6	6
Hematoma	2	2	–	–	–	4
Dehiscence	–	–	–	2	3	5
Malunion	–	–	1	–	2	3
Granuloma	–	1	–	–	–	1
Total	6	66			60	132

Values are presented as number

The total number of patients who experienced complications was 126, but 4 patients had experienced 2 complications and 1 patient had experienced 3

*For nerve damage, it was difficult to determine the specific region in the midfacial area

**Table 3:** Postoperative complications according to the fracture cause

Complication	Ground	Assault	Sports	TA	Object	Fall	Total
Nerve damage	46	17	4	21	2	5	95
Infection	5	7	–	5	–	1	18
Malocclusion	–	2	1	3	–	–	6
Hematoma	1	2	1	–	–	–	4
Dehiscence	1	2	–	2	–	–	5
Malunion	1	–	–	2	–	–	3
Granuloma	1	–	–	–	–	–	1
Total	55	30	6	33	2	6	132

Values are presented as number

The total number of patients who experienced complications was 126, but 4 patients had experienced 2 complications and 1 patient had experienced 3

*Ground ground accident, TA* traffic accident, Object hit by flying object

**Table 4:** Results of multivariable logistic regression analysis for factors affecting the occurrence of postoperative complications

Risk factors	Odds ratio	95% CI	*p* value
Day*			.014
11th–20th	0.923	0.551–1.547	.762
21th–31th	1.697	1.042–2.763	.033
Fracture site^†^			.000
Solitary mandible	7.963	5.299–11.966	.000
Midface and mandible	9.188	3.343–25.256	.000
Cranial bone-related	1.534	0.188–12.522	.690
Cause^‡^			.005
Assault	0.852	0.526–1.379	.515
Sports	0.562	0.234–1.349	.197
TA	2.456	1.453–4.152	.001
Flying object	0.546	0.127–2.347	.416
Fall	1.198	0.453–3.166	.716

Hosmer–Lemeshow goodness-of-fit test, *p* = .935. Akaike information criterion, 836.547

*CI* confidence interval, *TA* traffic accident

*Compared to days 1–10

^†^compared to solitary fracture of the midface

^‡^compared to ground accident

## Discussion

The present study involved a retrospective analysis of medical records of patients with craniofacial fractures who visited the departments of emergency, dentistry, otorhinolaryngology, plastic surgery, and neurosurgery over a period of 8 years, in order to update the data on craniofacial fractures in the capital of South Korea and use them as guidelines for the treatment and policy for craniofacial fractures.

There were more male patients than female patients in all age groups. This can be explained by the fact that men are more exposed than women to activities such as drinking, driving, and assault. These results are consistent with previous findings indicating a 3.6- to 5.4-fold higher incidence of fracture among men than among women [7–9]. In the present study, patients in their twenties showed the highest incidence of fracture, with the frequency gradually decreasing with increase in age. This result is also consistent with the findings of many previous studies [7, 8]. People in the age group of 20–29 years are often exposed to trauma because of their high levels of physical and social activities, which entail a high risk of fracture. In addition, in a study by Atisha et al., 89.7% of patients with facial fractures were below 65 years of age, which is similar to the proportion observed in the present study [10].

In the present study, the proportion of patients from 2014 to 2017 showed a tendency to decrease compared to that from 2011 to 2013, with the smallest proportion of patients with fracture being recorded in 2016. In a study by Jeon et al., the number of patients with facial fracture trauma in the late 2000s was about 4.2 and 2.4 times higher than that in the 1980s and 1990s, respectively [11]. However, the present findings show that the number of patients with craniofacial fracture has decreased in recent years, which suggests that the incidence of fracture has not continued to increase. The number of patients with fracture was the highest in the middle trisection of each month and the lowest in the first trisection. This is consistent with the findings of Park et al. [12]. These results are related to the fact that, currently, Korean workers are generally paid after the first half of the month and that many social events are held after the receipt of salary. It is necessary to consider these factors when arranging trauma staff for the emergency room. In terms of weekly distribution, weekends had a higher number of patients than weekdays, although the difference was not statistically significant. However, Kwon et al. reported that facial fractures occur most frequently on Sundays [13].

In the present study, most patients had fractures localized to the midface. Arslan et al. reported a 6.1:1 ratio of midfacial and mandibular fractures in their study, which is similar to the present results [14]. In contrast, Motamedi et al. reported a higher incidence of mandibular fractures (65.1%) than midfacial fractures in their study [8]. In the present study, the nasal bone was the most common site of fracture, followed by the orbital wall and zygomatic arch. On the other hand, Motamedi et al. reported the highest frequency of midfacial fractures in the zygomatic arch [8]. In mandibular fractures, the mandibular symphysis was the most frequent fracture site, followed by the mandibular angle, condyle, and body. This is in agreement with the findings of Motamedi et al. but inconsistent with the results of Morris et al. and James et al. [7, 8, 15]. Because of regional, social, economic, and cultural differences, the results in this regard vary from study to study. In addition, the results have also been observed to vary depending on the institution where the study was conducted. The midface is the most common fracture site in case of fractures requiring plastic surgery, while the mandible is the most common fracture site in dentistry [11]. Patients with cranial bone fracture were rare in the present study. Among skull fractures, frontal bone fracture was the most common type (50%), which coincides with the previous findings [16].

The number of fractures on the left side was 1.25 times greater than that on the right side in the present study. This trend seems to be related to the fact that assault is a considerably frequent cause of accidents and that there are more right-handed people than left-handed people in the general population in South Korea. The reason for the high incidence of bilateral fractures in the mandibular symphysis in the present study is that a case in which the fracture line ran across the mandibular midline was designated as a bilateral fracture. Patients with single-site fracture were 4.1 times more frequent than those with multiple-site fractures. This is attributable to the present data being greatly affected by the high incidence of nasal bone fracture, which had the highest proportion among the overall and single-site fractures. Lee et al. and Ribeiro et al. reported single-site fracture rates of 75.2% and 67.7%, respectively, in their studies; these results are similar to the present findings [17, 18]. However, excluding the instances of single or simultaneous nasal fracture, the rate of multiple fractures in the present study was about 40%. In terms of individual fracture sites, the rate of simultaneous fracture was greater than that of single fracture at all sites except the mandibular condyle and cranial bone. Additionally, cases of zygomatic arch and maxillary wall fracture were frequently associated with zygomaticomaxillary complex fracture and showed multiple-site fractures in 79.0% and 90.5% of the cases, respectively. In the present study, 41% of the patients with mandibular fracture exhibited multiple-site fractures. Similarly, in the study by Morris et al., over 50% of mandibular fractures were multiple-site fractures [7]. In addition, the facial surface is considered to have a high probability of multiple-site fracture because many bones are arranged in a relatively small area. Therefore, it is desirable to start with the assumption of multiple-site fractures for adequate diagnosis of facial fractures.

In previous studies, the most common cause of facial fracture was traffic accident [8, 17, 19]. However, recent advances in technology have reduced the number of traffic accidents and increased the safety of drivers and passengers. In recent studies, the most common cause of facial fracture has been assault or fall [9, 10, 14, 18, 19]. In the present study, ground accident was the most common cause of facial fracture, followed by assault. On the other hand, only 10% of fractures were caused by traffic accidents. Therefore, the present results are consistent with those of the recent studies. The frequency of ground accidents was high (about 65%) among children below 10 years of age and among the elderly; it was the lowest among patients in their twenties and showed a tendency to increase with age. Assault was the most common cause of fracture among teenagers and patients in their twenties. In addition, the proportion of sports-related fractures was the highest among teenagers, with the frequency decreasing with increase in age; the frequency of sports-related fractures was very low among patients aged 40 and above. This tendency can be explained by the fact that the younger the age, the greater the physical and interpersonal activity. Approximately 20.0% of all patients and 22.1% and 30.0% of patients with fractures due to ground accident and assault, respectively, were under the influence of alcohol, suggesting that there is a significant association between alcohol consumption and incidence of facial fracture. These results are consistent with those of previous studies by Lee et al. and Arslan et al. [14, 18].

Although there have been few studies on the relationship between the cause and site of injury, the present results have confirmed that there might be specific causes for injury in specific areas. For example, relative to other fractures, nasal bone fracture was more related to ground accident, assault, sports activity, and flying objects, while zygomatic arch and maxillary wall fractures were more related to fall and traffic accidents. Therefore, in sports activities, it is necessary to manufacture and wear helmets that take into consideration the protection of the nasal area; additionally, in automobile design, it is necessary to design airbags that can protect the zygomaticomaxillary complex.

During the bone healing process after a fracture, the proliferative phase begins 3 days after injury, where the woven bone is replaced by mature bone, resulting in a subsequent increase in mechanical strength by approximately 3 weeks after the fracture [20]. Therefore, it is ideal to perform treatment within 2 weeks after injury in order to achieve proper reduction without intentional fracture. In addition, if a muscle or nerve is damaged or stressed by the fracture, treatment at the earliest opportunity ensures good prognosis [13]. In the present study, the average interval from injury to treatment was about 6.5 days; while 68.6% of the patients were treated within a week of injury, 94.6% were treated within 2 weeks. In other words, most patients received treatment within a proper period of time.

In this study, postoperative complications were observed in 6.1% of the patients. The last trisection of a month, mandible-related facial fractures, and traffic accidents were associated with postoperative complications. With regard to the last trisection, as described above, social meetings and alcohol consumption are higher at this time than at the beginning of the month. It is, therefore, thought that not only the frequency of trauma but also the severity of damage is higher at the end of the month than at the beginning, which is related to the higher incidence of postoperative complications during the last trisection. In regard to the mandible, the relationship of mandible-related fractures with nerve injury can be considered. In fact, the most frequent complication in the present study was nerve damage. This can be explained by the fact that the inferior alveolar nerve is located within the mandible and is vulnerable to damage. In case of traffic accidents, because they typically involve high-velocity blunt trauma, it is thought that postoperative complications are more common in such accidents than in those involving low-velocity trauma. In this study, the most common postoperative complication apart from nerve damage was infection, most instances of which occurred in the mandible and maxillary wall, probably because of the higher possibility of infection in oral wounds than in skin wounds. Motamedi et al. reported nerve damage as the most common postoperative complication in their study, with about 16% of their patients experiencing inferior alveolar nerve damage, which is similar to the present results [8]. In a study by Brasileiro and Passeri, the most common postoperative complications related to facial fracture were infection and malocclusion, with most infections occurring in the mandible (31 of 38 patients) [21].

Because this study was conducted with data from only one hospital, the generalizability of the present findings might be low. However, many previous studies have included patients with facial fracture who visited a specific department for a certain period of time and, thus, reported a greater tendency for fracture in specific areas. In contrast to previous studies, the present study is more representative of facial fracture patterns because it included all patients with facial fracture who actually underwent surgery over the last 8 years in all of the departments that treated facial fracture. In addition, in this study, surgery was performed not by a single surgeon but by various surgeons from various departments. Moreover, this study excluded patients with a facial fracture who received conservative treatment. However, the decision-making process for conservative or surgical treatment might have varied from clinician to clinician; therefore, one of the study limitations is that no correction was made in this regard.

## Conclusions

In this study, craniofacial fractures were relatively prominent among males aged 10 to 39 years. The present findings indicated that a greater emergency room workforce is necessary during the middle 10 days of a month. The midfacial area, which had a relatively high proportion of facial fractures, should be preferentially evaluated. Excluding patients with nasal bone fractures, nearly half of the patients in the present study had experienced multiple fractures. Nasal bone fractures had a greater relationship with sports than with other causes of fracture; therefore, it is necessary to protect the nasal area during sports activities. Furthermore, zygomatic arch and maxillary wall fractures had a greater causal relationship with traffic accidents than with other causes, which should be reflected in improved protective mechanisms for drivers of automobiles. Fractures were associated with alcohol intoxication, especially in cases of personal assault. The last trisection of the month, mandible or panfacial fractures, and traffic accidents were related to a high incidence of postoperative complications. Surgeons should consider these factors when dealing with a craniofacial fracture.

## Additional file

Additional file 1: **Table S1.** Specific fracture region in each patient. **Table S2.** Results of univariable logistic regression analysis for factors affecting the occurrence of postoperative complications. (DOCX 22 kb)
