# Supplementary material for: Fracture patterns and causes in the craniofacial region: an 8-year review of 2076 patients
Source: Maxillofac Plast Reconstr Surg. 2018 Oct 15;40(1):29. doi: 10.1186/s40902-018-0168-y (PMC6186527; doi:10.1186/s40902-018-0168-y)
Supplement: Supplementary file 1 — Table S1. Specific fracture region in each patient. Table S2. Results of univariable logistic regression analysis for factors affecting the occurrence of postoperative complications. (DOCX 22 kb) [file 40902_2018_168_MOESM1_ESM.docx]

**Table S1.** Specific fracture region in each patient

| **Region** | **Number of patients** | **Region** | **Number of patients** |
| --- | --- | --- | --- |
| Solitary nasal bone | 1199 (57.8) | S+B+C | 3 (0.1) |
| Solitary orbital wall | 228 (11.0) | A+C | 1 (0.0) |
| Solitary zygomatic arch | 56 (2.7) | S+B+A | 1 (0.0) |
| Solitary maxillary wall | 24 (1.2) | M+S | 2 (0.1) |
| Solitary symphysis | 47 (2.3) | O+M+S | 1 (0.0) |
| Solitary angle | 48 (2.3) | Z+S+ | 1 (0.0) |
| Solitary condyle | 44 (2.1) | N+O+M+S | 1 (0.0) |
| Solitary body | 12 (0.6) | O+Z+M+B+A | 1 (0.0) |
| Solitary cranial bone | 9 (0.4) | N+B | 1 (0.0) |
| O+Z+M | 146 (7.0) | N+S+A | 1 (0.0) |
| N+O | 73 (3.5) | N+C | 1 (0.0) |
| Z+M | 25 (1.2) | N+O+B | 1 (0.0) |
| N+O+Z+M | 15 (0.7) | O+Z+M+S+B+A | 1 (0.0) |
| N+M | 12 (0.6) | N+O+Z+N+S | 1 (0.0) |
| O+Z | 10 (0.5) | O+Z+M+S | 1 (0.0) |
| O+M | 10 (0.5) | O+Z+M+SC | 1 (0.0) |
| N+O+M | 6 (0.3) | Z+M+S+A | 1 (0.0) |
| N+Z | 2 (0.1) | O+Z+M+S+C | 1 (0.0) |
| N+O+Z | 1 (0.0) | N+O+Z+M+B | 1 (0.0) |
| S+A | 37 (1.8) | N+O+Z+M+S+A | 1 (0.0) |
| S+C | 29 (1.4) | O+Z+M+B | 1 (0.0) |
| B+A | 9 (0.4) | N+Cr | 1 (0.0) |
| S+B | 4 (0.2) | O+Z+M+Cr | 1 (0.0) |
| B+C | 3 (0.1) | S+Cr | 1 (0.0) |

Symphysis, mandibular symphysis or parasymphysis; Angle, mandibular angle; Condyle, mandibular condyle; Body, mandibular body; O, orbital wall; Z, zygomatic arch; M, maxillary wall; N, nasal bone; S, mandibular symphysis or parasymphysis; A, mandibular angle; C, mandibular condyle; B, mandibular body; Cr, cranial bone

Values are presented as number (%).

The sum of the percentage value does not equal 100% because of rounding.

**Table S2.** Results of univariable logistic regression analysis for factors affecting the occurrence of postoperative complication

| Risk factors | Odds ratio | 95% CI | P value |
| --- | --- | --- | --- |
| Sex | .847 | 0.547-1.312 | .457 |
| Age | 1.009 | 0.999-1.020 | .075 |
| Diabetes mellitus | .645 | 0.200-2.097 | .463 |
| Smoking | 1.464 | 1.019-2.102 | .039 |
| Alcohol intoxication | 1.098 | 0.707-1.704 | .677 |
| Day of the week^*^ |  |  | .164 |
| Tuesday | .418 | 0.183-0.955 | .039 |
| Wednesday | .893 | 0.441-1.807 | .753 |
| Thursday | 1.289 | 0.658-2.523 | .459 |
| Friday | .837 | 0.418-1.675 | .615 |
| Saturday | .899 | 0.465-1.741 | .753 |
| Sunday | 1.185 | 0.629-2.234 | .599 |
| Day^†^ |  |  | .029 |
| 11^th^-20^th^ | 1.129 | 0.691-1.844 | .628 |
| 21^th^-31^th^ | 1.741 | 1.098-2.760 | .018 |
| Time from injury to treatment | .988 | 0.954-1.024 | .513 |
| Duration of admission | 1.024 | 1.011-1.037 | .000 |
| Fracture site^‡^ |  |  | .000 |
| Solitary mandible | 7.678 | 5.182-11.376 | .000 |
| Midface and mandible | 15.633 | 5.959-41.013 | .000 |
| Cranial bone-related | 2.436 | 0.310-19.155 | .397 |
| Cause^§^ |  |  | .000 |
| Assault | 0.768 | 0.483-1.221 | .264 |
| Sports | 0.416 | 0.177-0.979 | .045 |
| TA | 2.436 | 1.508-3.935 | .000 |
| Flying object | 0.412 | 0.098-1.724 | .225 |
| Fall | 1.611 | 0.664-3.907 | .291 |

CI, confidence interval; TA, traffic accident

*Compared to Monday; †compared to days 1-10, ‡compared to solitary fracture of maxilla; §compared to ground accident
